# *C. elegans* maximum velocity correlates with healthspan and is maintained in worms with an insulin receptor mutation

**DOI:** 10.1038/ncomms9919

**Published:** 2015-11-20

**Authors:** Jeong-Hoon Hahm, Sunhee Kim, Race DiLoreto, Cheng Shi, Seung-Jae V. Lee, Coleen T. Murphy, Hong Gil Nam

**Affiliations:** 1 Center for Plant Aging Research, Institute for Basic Science, Daegu 42988, Republic of Korea; 2 Department of Molecular Biology, Lewis-Sigler Institute for Integrative Genomics, Princeton University, 148 Carl Icahn Laboratory, Washington Road, Princeton, New Jersey 08544, USA; 3 Department of Life Sciences and School of Interdisciplinary Bioscience and Bioengineering, Pohang University of Science and Technology, Pohang, Gyeongbuk 790-784, Republic of Korea; 4 Department of New Biology, DGIST, Daegu 42988, Republic of Korea

## Abstract

Ageing is marked by physical decline. *Caenorhabditis elegans* is a valuable model for identifying genetic regulatory mechanisms of ageing and longevity. Here we report a simple method to assess *C. elegans*’ maximum physical ability based on the worms’ maximum movement velocity. We show maximum velocity declines with age, correlates well with longevity, accurately reports movement ability and, if measured in mid-adulthood, is predictive of maximal lifespan. Contrary to recent findings, we observe that maximum velocity of worm with mutations in *daf-2(e1370)* insulin/IGF-1 signalling scales with lifespan. Because of increased odorant receptor expression, *daf-2(e1370)* mutants prefer food over exploration, causing previous on-food motility assays to underestimate movement ability and, thus, worm health. Finally, a disease-burden analysis of published data reveals that the *daf-2(e1370)* mutation improves quality of life, and therefore combines lifespan extension with various signs of an increased healthspan.

## Abstract (web summary)

Increases in lifespan do not necessarily translate into prolonged healthspan. Here, the authors devise a simple metric, maximum velocity, to study ageing in *C. elegans* and, using this metric, show that reduced insulin signalling improves physical healthspan as well as worm lifespan.

## Graphical abstract

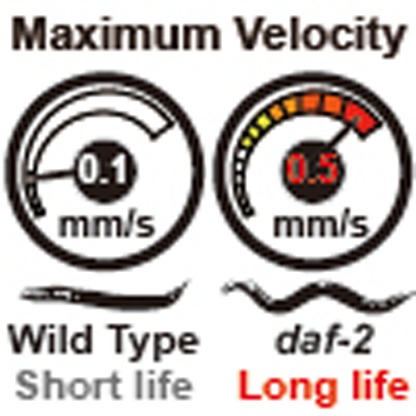

Ageing is an important risk factor for disease and is accompanied by the decline of many physiological characteristics, including physical performance, negatively affecting quality of life. Therefore, quality of life with age, not just longevity, is important for our ageing society. *Caenorhabditis elegans* has been a valuable model organism in revealing the genetic regulatory mechanisms of ageing and longevity^1^,^2^. One of the best-studied longevity pathways is the insulin/IGF-1 signalling (IIS) pathway, which regulates longevity in response to nutrient levels through its control of the activity of the FOXO transcription factor and its downstream targets^3^,^4^,^5^,^6^. Mutations of the DAF-2 insulin receptor, particularly the canonical allele *daf-2(e1370)*, double lifespan^3^ and extend the abilities to learn and remember, resist stress and infection, repair axons^7^, and suppress neuronal morphological defects^8^,^9^,^10^ with age. The components of the IIS pathway are well-conserved, and are linked to extreme longevity in humans^11^ as well. Recently, despite the fact that the *daf-2* mutation extends many physiological functions with age^7^,^8^,^9^,^10^, Bansal *et al*. concluded that *daf-2(e1370)* IIS mutants are less healthy than wild-type animals, disproportionately extending their ‘unhealthy’ lifetime^2^,^12^.

One of the most powerful tests of physical ability in elderly humans is the short physical performance battery (SPPB)^13^, an assessment of the maximum exercise capacity of lower extremities over a short period of time. The SPPB accurately predicts risk of disability^14^, length of hospital stay, and mortality over the year after hospital discharge^15^. The SPPB includes a short (4 m) walk to measure maximum gait speed. Like humans, *C. elegans* shows age-related decline in physical ability, which is manifested by reduced body movement^16^,^17^,^18^,^19^,^20^,^21^,^22^. We wondered whether a similar metric of maximum velocity (MV) in a short period (30 s) would be equally informative in *C. elegans*. We found that worms’ MV declines with age, correlates well with longevity, accurately reports movement ability, and importantly, is predictive of future longevity. *daf-2(e1370)* mutations extend MV and ‘healthspan’, scaling with lifespan^19^. Motility assays performed on bacteria underestimate *daf-2(e1370)* mutants’ movement ability due to their high *odr-10* levels and subsequent preference for food over exploration. We used human disease-burden models to assess *C. elegans* mutants’ quality of life; quality-adjusted reanalysis of data from previously published healthspan studies^2^,^19^ demonstrates that *daf-2(e1370)* mutants’ healthspan correlates with longevity extension, and in terms of disease-burden analysis, improves quality of life. The IIS pathway mutation *daf-2(e1370)* coordinates the extension of lifespan with many aspects of healthspan, providing valuable insight into mechanisms that extend functional health with age.

## Results

### MV correlates with lifespan

To mimic the SPPB test used to assess health in elderly humans, we measured worms’ MV over 30 s on an unseeded nematode growth medium (NGM) plate each day of adulthood (Supplementary Fig. 1). Our assay differs from previous motility assays in its brevity (30 s versus 5 min (ref. 21) or longer^23^), longitudinal testing of individual worms rather than populations, and measurement on plates without bacteria^2^. MV appears to not simply be a response to harsh touch, as harsh-touch mutants behave like wild-type worms in the assay, and the MV is distributed over the entire 30 s window for all animals (Supplementary Fig. 2).

**Figure 1:**
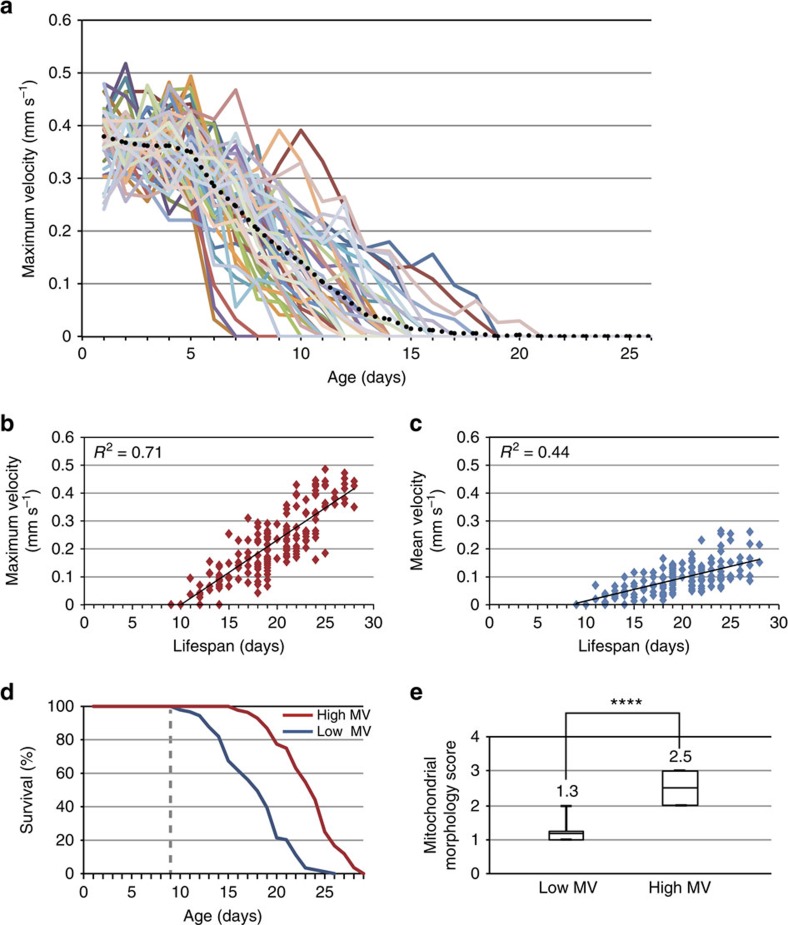
MV is correlated with longevity. (**a**) Age-dependent changes in the MV of 52 individual wild-type worms. Black dots represent the average value at each age. (**b**,**c**) Correlation between MV and lifespan (**b**) and correlation between mean velocity and lifespan (**c**) of individual worms. The MVs and mean velocities of individual worms were measured at day 9 of adulthood and their lifespans were measured thereafter. *r*^2^, coefficient of determination. *n*=173. (**d**) Survival curves of the low-MV (*n*=89) and high-MV (*n*=84) group worms. The groups were divided at day 9 of adulthood (grey dotted line) and the survivorship of worms in each group was measured thereafter. (**e**) Correlation of MV with mitochondrial morphology at day 9 of adulthood. Scores of mitochondrial morphology of low-MV and high-MV groups were 1.3±0.5 and 2.5±0.5, respectively (*n*=27). The bottom and top of the box and whiskers are the first and third quartiles, and the band inside the box is the average. Significance was determined using a two-tailed, unpaired *t*-test. ****P<0.0001.

To assess age-associated decline of physical performance, we measured MV of 52 individual wild-type worms each day of their lifespan (Fig. 1a). We found that motility decreased with age (Supplementary Fig. 3), with MV declining earlier and more drastically than mean velocity (Supplementary Fig. 3). During the early phase of adulthood (day 1–5), MV of individual worms was maintained in the range of 0.22–0.52 mm s^−1^. However, from day 6 onwards, all worms showed a decline in MV with age.

We grouped worms into high (≥0.22 mm s^−1^) and low (≤0.21 mm s^−1^) MVs; the criterion was based on the minimum MV at day 1 of adulthood. The population of the low-MV group increased almost exponentially, reaching 54.6% by day 9 of adulthood (Supplementary Fig. 4). We then examined the correlation between MV of worms at day 9 of adulthood (midlife) and their lifespan thereafter. Day 9 MV correlated well with lifespan (coefficient of determination (*r*^2^)=0.71), better than the correlation between lifespan and mean velocity (*r*^2^=0.44; Fig. 1b,c), thrashing rate (*r*^2^=0.18; Supplementary Fig. 5), or pharyngeal pumping (*r*^2^=0.65; Supplementary Fig. 5). Remarkably, the median lifespan of worms in the day 9 high-MV group (23±3.2 days) was 35.3% longer than that of the worms in the day 9 low-MV group (17±3.6 days; Fig. 1d). Thus, MV of wild-type worms at day 9 of adulthood is a reliable predictor of longevity thereafter.

In humans, mitochondrial defects are associated with ageing of skeletal muscle and are correlated with reduced physical strength in the elderly^24^,^25^,^26^. Young *C. elegans*’ mitochondria are tubular^27^; however, gradual fragmentation with age results in sparse, globular mitochondria (Supplementary Fig. 6). At day 9 of adulthood, worms in the low-MV range had very fragmented mitochondria, while the high-MV range group’s mitochondria were significantly less damaged at the same age (Fig. 1e and Supplementary Fig. 7). In addition, expression of the protective lifespan biomarker *sod-3* (ref. 28) was 1.7-fold higher in the high-MV group than in the low-MV group (Supplementary Fig. 8). These data show that MV correlates with two age-associated physiological parameters, and suggest that mechanisms determining MV might also regulate mitochondrial integrity maintenance and stress protection gene expression.

### *daf-2(e1370)* mutation extends healthspan

**Figure 2:**
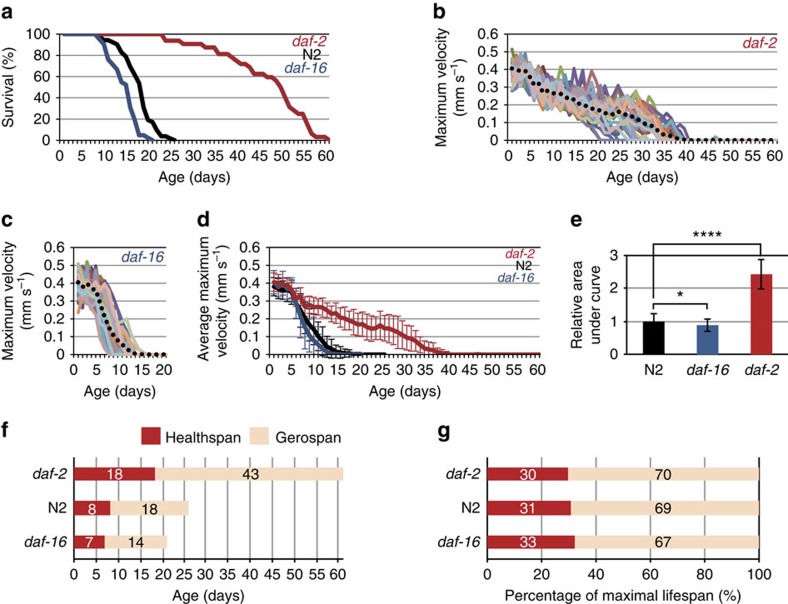
Correlation of MV with lifespan in *daf-2* mutants. (**a**) Age-associated change of survivorship in N2 (wild type) (*n*=52), *daf-2(e1370)* (*n*=32) and *daf-16(mu86)* (*n*=42) worms. (**b**,**c**) Age-associated change of MVs in *daf-2* (**b**) and *daf-16* mutant worms (**c**). (**d**) Age-associated change of average MVs in N2 (*n*=52), *daf-2* (*n*=32) and *daf-16* (*n*=42) mutant worms. Error bars represent s.d. (**e**) A cumulative difference in physical performance in N2 and *daf-16* and *daf-2* mutant worms as indicated by area under curves in **d**. The relative values of area under curves are 1.0±0.2, 0.9±0.2 and 2.4±0.4 for N2, *daf-16* and *daf-2* worms, respectively. (**f**) Comparison of healthspan and gerospan in N2, *daf-16* and *daf-2* worms. Healthspan was defined as ‘the period during which the worm showed >50% maximal functional capacity of wild type’^2^. Gerospan was defined as ‘the period during which the worm showed <50% maximal functional capacity of wild type’^2^. (**g**) The healthspan-to-gerospan ratio normalized to their maximal lifespan in N2, *daf-16* and *daf-2* worms.

We next assessed the MV of long-lived *daf-2(e1370)* insulin/IGF-1 receptor mutants and short-lived mutants of the DAF-16 FOXO transcription factor (*daf-16(mu86))*, which is required for *daf-2* mutants’ longevity^3^,^4^,^29^ (Fig. 2a). *daf-2* animals exhibited a higher MV with age, particularly from day 10 onwards (Fig. 2b–d). At day 26 of adulthood, when all the wild-type worms had died, *daf-2* mutants still maintained on average 36% of MV (Fig. 2d). We examined the correlation of MV on each day with the lifespan for the worms in the longitudinally tracked cohort (Figs 1a and 2b,c). The correlation between MV and lifespan is best on day 9 for N2 worms, day 23 for *daf-2* and day 10 for *daf-16* (Supplementary Fig. 9); in all cases, the best correlation between MV and lifespan occurs within 1 day of the first death in the cohort. We integrated the area under the MV curve to serve as an indicator of overall physical performance; *daf-2* mutants showed a 2.4-fold increase over wild type, and *daf-16* mutants showed slightly lower physical performance (Fig. 2e). Thus, *daf-2(e1370)* mutation extends physical ability with age.

We then used MV to determine the ‘healthspan’ (defined as the period with >50% of maximal activity^2^) and ‘gerospan’ (period with <50% maximal activity^2^) ratios of wild-type, *daf-16(mu86)*, and *daf-2(e1370)* mutant worms, in the same manner as Bansal *et al*.^2^. While this type of analysis does not include any error calculation, our results suggest that *daf-2* mutants increased healthspan more than twofold compared with wild-type and *daf-16* mutants (Fig. 2f), and the resulting normalized healthspan-to-gerospan ratios were similar among the three strains (Fig. 2g). Thus, *daf-2(e1370)* mutation extends both lifespan and healthspan to a similar extent, without proportionally extending the unhealthy part of life, in contrast to Bansal *et al*.^2^ conclusions.

### *odr-10* levels determine on-food motility rates

**Figure 3:**
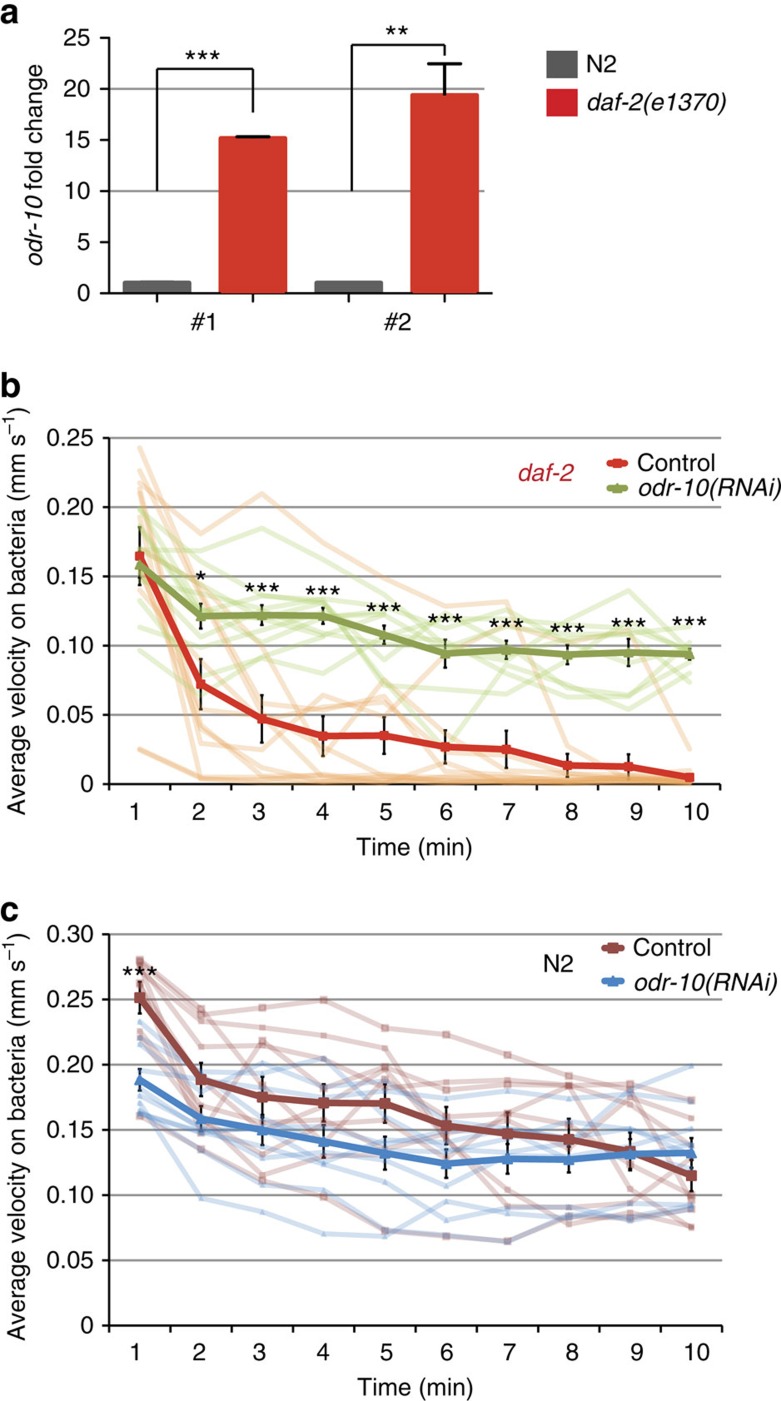
Motility on bacteria measures food preference rather than ability to move. (**a**) Two sets of primers (#1 and #2) were used to measure the expression of *odr-10* by qRT-PCR in N2 and *daf-2* mutants. For each primer set, *odr-10* expression in wild type was normalized to 1. *odr-10* expression in *daf-2* mutants increased by 15.2±0.2-fold and 19.4±3.1-fold, respectively. (**b**) Reduction of *odr-10* levels by RNAi increased *daf-2* motility on bacteria significantly. *daf-2* on OP50: 0.04±0.03 mm s^−1^; *daf-2; odr-10(RNAi)*: 0.11±0.01 mm s^−1^ (**c**) wild-type worms’ motility on bacteria was not significantly affected by reduction of *odr-10*: N2 worms on OP50: 0.17±0.03 mm s^−1^; N2 on *odr-10(RNAi):* 0.14±0.03 mm s^−1^. *n*=3, *P<0.05, **P<0.01, ***P<0.001, ****P<0.0001; unpaired *t*-test.

We were concerned that differences in motility assays may have led to discrepancies with the previous report^2^. Our assay measures MV on plates without bacteria, while Bansal *et al*.^2^ measured the distance worms moved on bacteria. We wondered whether *daf-2*’s lower motility on food might not reflect its ability to move, but rather its preference for food over exploration. Food-seeking preference is determined by levels of the odorant receptor *odr-10* (ref. 30); high levels of *odr-10* suppress food-exploration behaviour^30^. Ryan *et al*.^30^ showed that *odr-10*, despite the fact that it is only expressed in the AWA neurons and primarily detects diacetyl, plays a major role in food detection and is the primary factor in determining the decision of males to explore (mate search) rather than feed. Indeed, we found that the expression of *odr-10*, which contains a DAF-16-binding element 809 bp upstream of its start site, is elevated 15–20-fold in *daf-2(e1370)* mutants (Fig. 3a). Furthermore, we observed that reduction of *odr-10* levels dramatically increases *daf-2*’s motility on bacteria: unlike control-treated animals, which stop moving after 1.5–3 min when placed onto bacteria, *daf-2(e1370);odr-10(RNAi*) worms continue to move constantly over a 10 min assay period (Fig. 3b), and reduction of wild-type worms’ *odr-10* levels has no significant effect (Fig. 3c and Supplementary Movies 1, 2, 3). (Interestingly, knockdown of other genes involved in odour detection, including *odr-3*, *odr-7* and *osm-9*, did not have the same effect on *daf-2(e1370)* motility (Supplementary Fig. 10).) Therefore, motility can only be measured accurately off food, because movement on bacteria measures food-seeking rather than ability to move.

### *daf-2(e1370)* mutation increases quality of life

**Figure 4:**
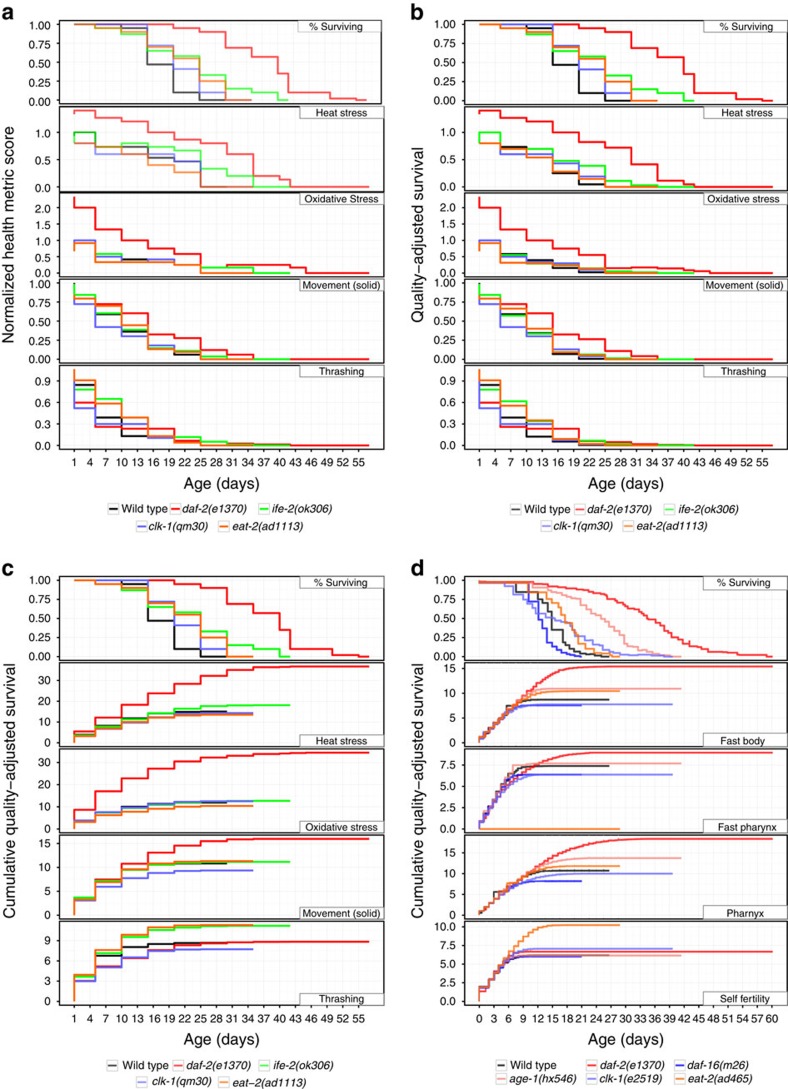
Quality-adjusted health metrics. *daf-2* mutants are healthier than wild type. (**a**) Quality-adjusted lifespan curves for the health metrics observed by Bansal *et al*.^2^, with each metric normalized to the maximum value of the wild type. (**b**) The quality-adjusted lifespan curve is the survival rate multiplied by the normalized health measurement. (**c**) Visualizing the cumulative area under the quality-adjusted lifespan curve at each time point shows that *daf-2* mutants have higher lifespan quality over most measured health metrics. (**d**) Analysis of additional healthspan data measured by Huang *et al*.^19^ shows a similar increase in total lifespan quality of *daf-2* mutants over wild type.

As MV is only one metric of healthspan, we reanalysed the entire Bansal *et al*.^2^ healthspan data set. (The same *daf-2* allele, *e1370*, was used in their studies, as well.) In our analysis, we normalized each health metric at each time point to the maximum score of wild type to directly compare performance levels (Fig. 4a). This reanalysis shows that *daf-2* mutants were healthier than wild type in all metrics except thrashing in liquid. However, previous thrashing measurements of *daf-2* mutants^31^ and another long-lived IIS mutant, *age-1* (ref. 32), demonstrate that these IIS mutants maintain thrashing activity with age better than wild type.

We wondered whether cost-utility (disease burden) methods developed to assess quality of life^33^ (which are typically used to make cost assessments of human disease treatment options) could be applied to these healthspan analyses of *C. elegans*. To do so, we combined information from the lifespan curve and health measurements reported by Bansal *et al*. to obtain a ‘quality-adjusted lifespan’ curve, according to the method of Zeckhauser and Shepard^33^. We multiplied the lifespan curve by the normalized healthspan curve, producing a curve that describes both the declining health of the animal and the declining survival of the population (Fig. 4b). By calculating the area under the curve (AUC) of the quality-adjusted lifespan, we can visualize total quality of life, taking into account both survival and health (Fig. 4c; Methods section). Reanalysis of the Bansal *et al*.^2^ data using this approach suggests that while not all longevity mutants have a commensurate increase in life quality (in fact, some longevity mutants, such as *clk-1*, exhibit lower total quality, as Bansal *et al*. reported), increased health and extended lifespan result in a higher total quality for *daf-2(e1370)* mutants. (We note that the cumulative quality-adjusted score for thrashing is equivalent for *daf-2(e1370)* and wild type, but that thrashing has little correlation overall with longevity (Supplementary Fig. 5).) We reached a similar conclusion upon reanalysis of the data from Huang *et al*.^19^, which also measured several healthspan parameters (Fig. 4d and Supplementary Fig. 11a,b). In addition, applying this analysis to our own MV data shows the same dramatic increase in *daf-2(e1370)* lifespan quality (Supplementary Fig. 11c–e). Thus it appears from multiple analyses of healthspan that *daf-2(e1370)* mutants have a higher quality of life than wild-type worms.

## Discussion

Here we have modelled the SPPB assessment of physical ability in elderly humans in worms, using a simple assay of *C. elegans*’ maximum ability to move over a short period. MV correlates well with age; in fact, MV performance at midlife (day 9) can predict the remaining lifespan of the individual. Our analysis also reveals that other widely used healthspan metrics are less correlated with lifespan; thrashing is a particularly poor correlate (*r*^2^=0.18), while pumping has a good correlation (*r*^2^=0.65) but shows highly irregular patterns (Supplementary Fig. 5). Thus, MV is a powerful healthspan proxy and may be the most informative metric of *C. elegans*’ health with age.

An unexpected benefit of this experimental design is that it is not confounded by the worms’ ongoing assessment of its bacterial food source. We discovered that *daf-2* has an inherently higher preference for food than exploration, likely due to its increased expression level of the *daf-2/daf-16* target *odr-10*; high levels of the ODR-10 odour receptor cause *daf-2* worms to prefer food over exploration, slowing its movement on bacteria. (Further, our data on other chemosensory genes suggest that *odr-10* is a particularly good indicator of food preference.) As this type of movement is not limited by ability, but rather by preference, it may be difficult to draw conclusions regarding healthspan from on-food motility assays.

Measurements that take into account both lifespan and healthiness are important in assessing quality of life with age, and the clinical implications of coordinated extension of lifespan and healthspan are immense. Not all lifespan interventions will improve health or total quality, as *clk-1* mutants demonstrate. In fact, several longevity mutants are less healthy overall than wild-type worms by different measurements, and it is important to note which mutants may have a long life with no health benefit. However, we find no evidence to support the claim by Bansal *et al*. that *daf-2(e1370)* mutants spend a greater fraction of life in a frail state; in fact, *daf-2* mutants demonstrate that healthspan and lifespan can be coordinately extended. From the analysis of our own data and that of published experiments, it seems clear that *daf-2* extends not only lifespan, but also many abilities with age. In addition to the standard *C. elegans*-specific ‘healthspan’ assays (heat stress, oxidative stress and movement), *daf-2* extends behaviours that are relevant for human decline with age, as well. This includes the abilities to learn and remember^34^, repair axons^7^, resist pathogenic infections^35^, suppress age-related neuronal morphological defects^8^,^9^,^10^ and neurodegenerative protein aggregation^36^, maintain neuromuscular junction activity^23^,^37^, and maintain high-quality oocytes with age^38^. Many of the downstream targets of the *daf-2* insulin signalling pathway^6^ that enable these functions have been identified, and many of these genes and the underlying mechanisms are conserved in mammals; therefore, the extended abilities that *daf-2* exhibits offers therapeutic target possibilities.

Defining ‘healthspan’ and ‘gerospan’ based on population maximum lifespans ignores the fact that quality is a continuum. Instead, our analysis takes into account the length of time an individual can expect to live, and how healthy that individual can expect to be with age. In the case of *daf-2(e1370)* mutants, quality of life is clearly superior. The mechanistic study of coordinated health and life extension might allow the development of therapeutics to remedy end-of-life problems or to compress morbidity, decreasing health costs. *C. elegans* IIS longevity mutants remain valuable tools in understanding the mechanisms by which we might achieve these goals.

## Methods

### Strains

The *C. elegans* strain, N2 Bristol, was used as the wild type. The N2 strain and the insulin/IGF-1 mutants, *daf-2(e1370)*, *daf-16(mu86)*, CF1553 *muIs84*[pAD76 *(sod-3::GFP)*] and PD4251 ccIs4251 I; *dpy-20(e1282)* IV were obtained from the *Caenorhabditis* Genetics Center.

### Measurement of worm MV

Synchronized worms were prepared by placing adult worms on a NGM plate and allowing them to lay eggs for 3 h. After removing the adult worms, each synchronized progeny was transferred to a single NGM plate and cultured to L4 stage. After 24 h, single worms were transferred to the physical assay plate and movements were recorded immediately. The assay conditions were as follows: 20 °C and ∼40% humidity, with no lid. The physical assay plate was prepared in the same manner as the NGM, but with no bacterial lawn. The recording system comprised a stereomicroscope (Nikon SMZ 745T), a charge-coupled device camera (TUCSEN TCH-5.0) and imaging software (TUCSEN ISCapture). A × 12.14 zoom lens was used to keep the worms within the field of view. Movement was recorded every 24 h throughout the lifespan of each worm. The recording period was 30 s at a rate of 30 frames per second. After recording, the worm was transferred to a fresh NGM plate (the ‘growth plate’ shown in Supplementary Fig. 1). The locomotion velocity was expressed as mm per second (the distance (mm) between displaced centroids per second). Recorded images were analysed by ImageJ and wrMTrck (plugin for ImageJ: www.phage.dk/plugins). The locomotion velocity data were imported into an Excel spreadsheet. The peak locomotion velocity observed in the 30 s period was used as the MV.

### Lifespan analysis

Lifespan was assessed on NGM plates at 20 °C. The number of live animals was scored every day until death. Lifespan was analysed by Oasis survival analysis software^39^.

### Pharyngeal pumping and thrashing rate decline assays

Synchronized single worms were observed every day. The number of contractions in the terminal bulb of pharynx was counted for 30 s (*n*=19). The change in the reciprocating motion of bending at the mid-body in S-basal medium was counted as a body bend. The number of body bends was counted for 30 s (*n*=20).

### Qualitative analysis of mitochondrial morphology

Morphological categories were defined according to Regmi *et al*.^27^: (1) images showing a majority of long interconnected mitochondrial networks were classified as tubular; (2) images showing a combination of interconnected mitochondrial networks along with some smaller fragmented mitochondria were classified as intermediate; (3) images showing a majority of short mitochondria were classified as fragmented; and (4) images showing sparse globular mitochondria were classified as very fragmented. We scored mitochondrial morphology as ‘1’ (very fragmented), ‘2’ (fragmented), ‘3’ (intermediate) and ‘4’ (tubular; Supplementary Fig. 7). Mitochondrial morphology was examined in PD4251 strains at day 9 of adulthood after MV was recorded. Worms were immobilized during imaging using 30 mM sodium azide. Imaging was performed using a microscope equipped with a C-Apochromat 40x/1.20W Korr FCS M27 and a photo-multiplier tube. Zen 2011software (black edition) was used to acquire fluorescent z stacks of individual animals (1 μm per slice).

### Observation of *sod-3* expression

*sod-3* expression was examined in CF1553 strain worms at day 9 of adulthood after MV was recorded. Imaging was performed using a microscope equipped with a C-Apochromat 10x/1.20W Korr FCS M27 and a photo-multiplier tube. Zen 2011software (black edition) was used to acquire fluorescent z stacks of individual animals (1 μm per slice). Worms were immobilized during imaging using 30 mM sodium azide.

### *odr-10* qRT-PCR measurements

Two sets of primers (#1 and #2) were used to measure the expression of *odr-10* by quantitative reverse transcription PCR (qRT-PCR) in N2 and *daf-2* mutants. For each primer set, *odr-10* expression in wild type was normalized to 1. *pmp-3* was used as a reference gene.

‘odr-10-qRT-1-r’ 5′-AACGGTGCGATGAACATGAC-3′

‘odr-10-qRT-1-f’ 5′-CTTCAGGTATCCCGATCTGAAACT-3′

‘odr-10-qRT-2-r’ 5′-TCAGATCGGGATACCTGAAGA-3′

‘odr-10-qRT-2-f’ 5′-TCTACTGCGGATATGCCACG-3′

Reference gene primers: *pmp-3*

Forward: 5′-AGTTCCGGTTGGATTGGTCC-3′

Reverse: 5′-CCAGCACGATAGAAGGCGAT-3′

### Quality-adjusted lifespan analysis

Quality metrics were taken from published work. Because the raw data from Bansal *et al*.^2^ were not made publicly available by the authors at time of submission of this paper, we extracted data from their figures using GetData Graph Digitizer. (No N or error information is available, however.) The lifespan (L(t)) (% surviving with age) and healthspan (H(t)) (health measurement with age) data were graphed using a left-hand step function. The curves were combined by multiplying the lifespan and healthspan curves together, with the value of the resulting quality-adjusted lifespan curve (Q(t)) being Q(t)=L(t) × H(t). The AUCs was calculated by taking the definite integral of this step function. The set of timepoints T is zero-indexed and consists of T={t_0_,t_1_,…,t_n_}, and we calculate the AUC as shown in equation (1).




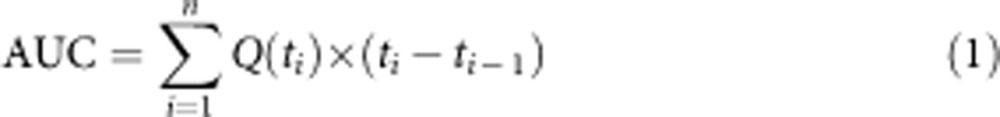




## Additional information

**How to cite this article:** Hahm, J.-H. *et al*. *C. elegans* maximum velocity correlates with healthspan and is maintained in worms with an insulin receptor mutation. *Nat. Commun.* 6:8919 doi: 10.1038/ncomms9919 (2015).

## Supplementary Material

Supplementary Information Supplementary Figures 1-11 and Supplementary Reference.

Supplementary Movie 1 A representative movie of odr-10 RNAi treated daf-2(e1370) worm's motility on food. Synchronized daf-2(e1370) eggs were transferred onto NGM plates seeded with odr-10 RNAi bacteria. On Day 2 of adulthood, individual worms were picked from the odr-10 RNAi bacteria plate and transferred to a fresh NGM plate with OP50 bacteria. Immediately after transfer, the movement of the worm was recorded continuously for 10 min. When the worm ran out of the field of view, the plate was relocated to chase the worm as noted by the sudden changes of the field of view in the movie. Note the continuous locomotion of the worm. The movie is 10x faster than real time. At least 10 worms were filmed for the statistical analysis of the locomotive activity.

Supplementary Movie 2 A representative movie of control daf-2(e1370) worm's motility on food. Synchronized eggs (the same population as in supplementary movie 1) were transferred onto NGM plates seeded with OP50 bacteria. On Day 2 of adulthood, individual worms were picked from a NGM plate and transferred to a fresh NGM plate with OP50 bacteria. Immediately after transfer, the movement of the worm was recorded continuously for 10 min. When the worm ran out of the field of view, the plate was relocated to chase the worm as noted by the sudden changes of the field of view in the movie. Note that the worm starts to show a low locomotive activity within 2 min in this case. The movie is 10x faster than real time. At least 10 worms were filmed for the statistical analysis of the locomotive activity.

Supplementary Movie 3 A representative movie of wild type (N2) worm's motility on food. N2 worms were grown on NGM plates seeded with OP50 bacteria. On Day 2 of adulthood, individual worms were picked from a NGM plate and transferred to a fresh NGM plate with OP50 bacteria. Immediately after transfer, the movement of the worm was recorded continuously for 10 min. When the worm ran out of the field of view, the plate was relocated to chase the worm as noted by the sudden changes of the field of view in the movie. Note the continuous locomotion of the worm. The movie is 10x faster than real time. At least 10 worms were filmed for the statistical analysis of the locomotive activity.
