# Supplementary material for: C. elegans maximum velocity correlates with healthspan and is maintained in worms with an insulin receptor mutation
Source: Nat Commun. 2015 Nov 20;6:8919. doi: 10.1038/ncomms9919 (PMC4656132; doi:10.1038/ncomms9919)
Supplement: Supplementary Information — Supplementary Figures 1-11 and Supplementary Reference. [file ncomms9919-s1.pdf]

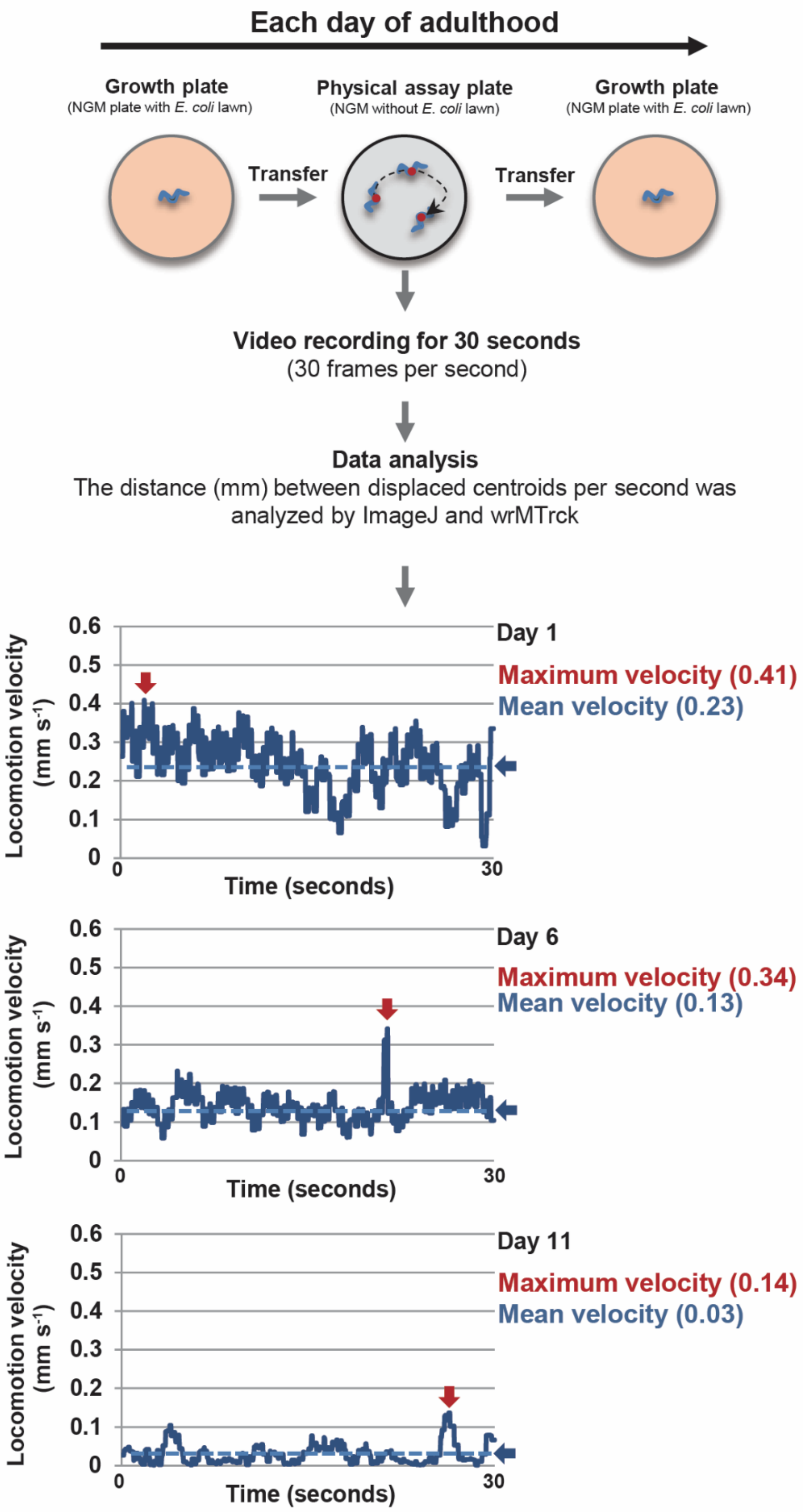
Each day of adulthood
Growth plate
(NGM plate with E. coli lawn)
Transfer
Physical assay plate
(NGM without E. coli lawn)
Transfer
Growth plate
(NGM plate with E. coli lawn)
Video recording for 30 seconds
(30 frames per second)
Data analysis
The distance (mm) between displaced centroids per second was analyzed by ImageJ and wrMTrck
Locomotion velocity (mm s-1)
Time (seconds)
Day 1
Maximum velocity (0.41)
Mean velocity (0.23)
Day 6
Maximum velocity (0.34)
Mean velocity (0.13)
Day 11
Maximum velocity (0.14)
Mean velocity (0.03)

Supplementary Figure 1: The assay scheme for measuring the age-associated decline of short physical performances in *C. elegans*.

Worms were grown on a growth plate covered with a bacterial lawn. When measuring the physical performance, the worms were transferred to a ‘physical assay plate’ with no bacterial lawn and their movement was recorded for 30 seconds. The red dots indicate the centroids used to follow the movement of the worms. ImageJ and wrMTrck were used to calculate the locomotion velocity, which was expressed as mm s^{-1} [distance (mm) between centroid displacements per second]. The velocity profiles of a single worm at the three different ages (Day 1, 6, and 11 of adulthood) are shown at the bottom. The MV and mean velocity at the three different ages are noted.

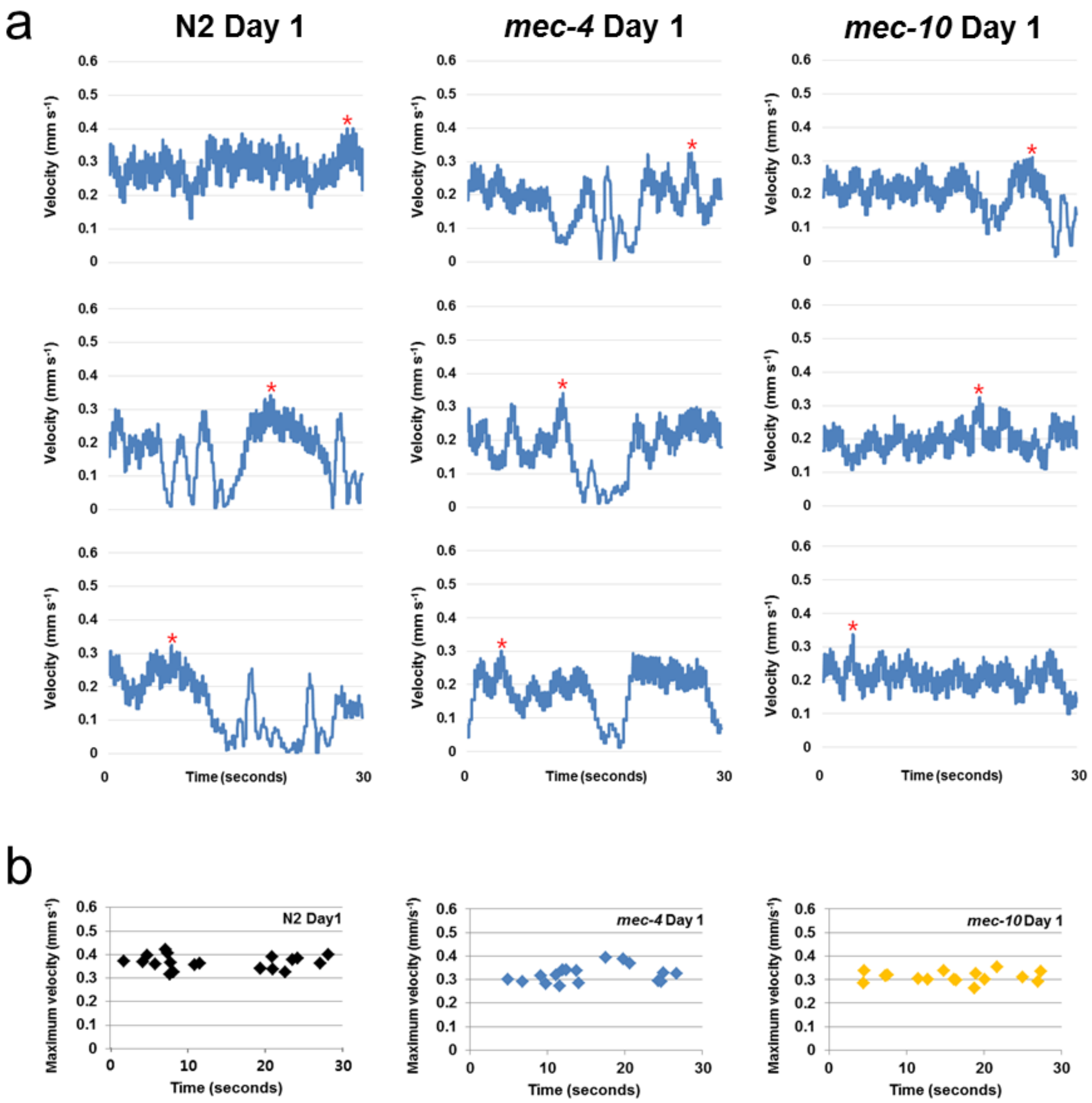

Supplementary Figure 2: Harsh-touch mutants do not behave differently from wild-type in the maximum velocity (MV) assay. (**a**) Mechanosensory mutants (*mec-4* and *muc-10*) show a velocity pattern similar to that of wild type in the MV assay. Red asterisk represents maximum velocity. (**b**) Maximum velocity appeared randomly on the 30 seconds interval of the MV assay in both wild type and mechanosensory mutants.

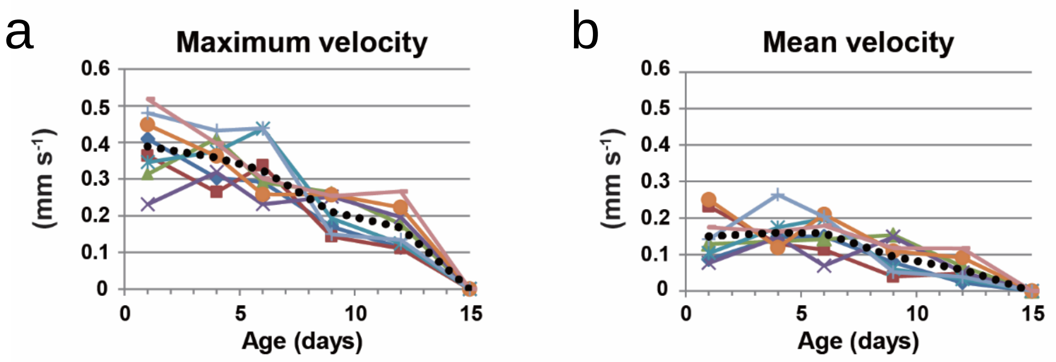

Supplementary Figure 3: Age-dependent changes of maximum (a) and mean (b) velocity of individual worms. Black dots represent the average value at each age (*n* = 8).

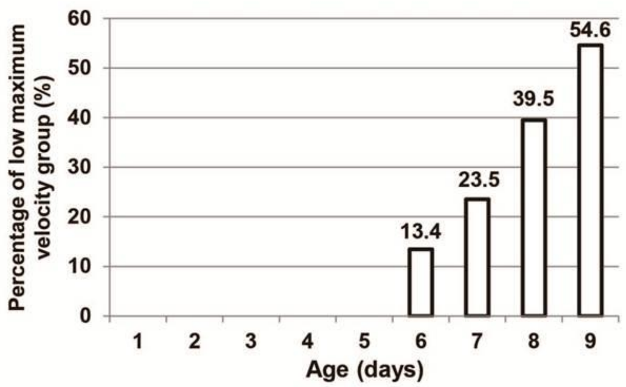

Supplementary Figure 4: Age-associated increase of percentage of low MV group (*n* = 119).

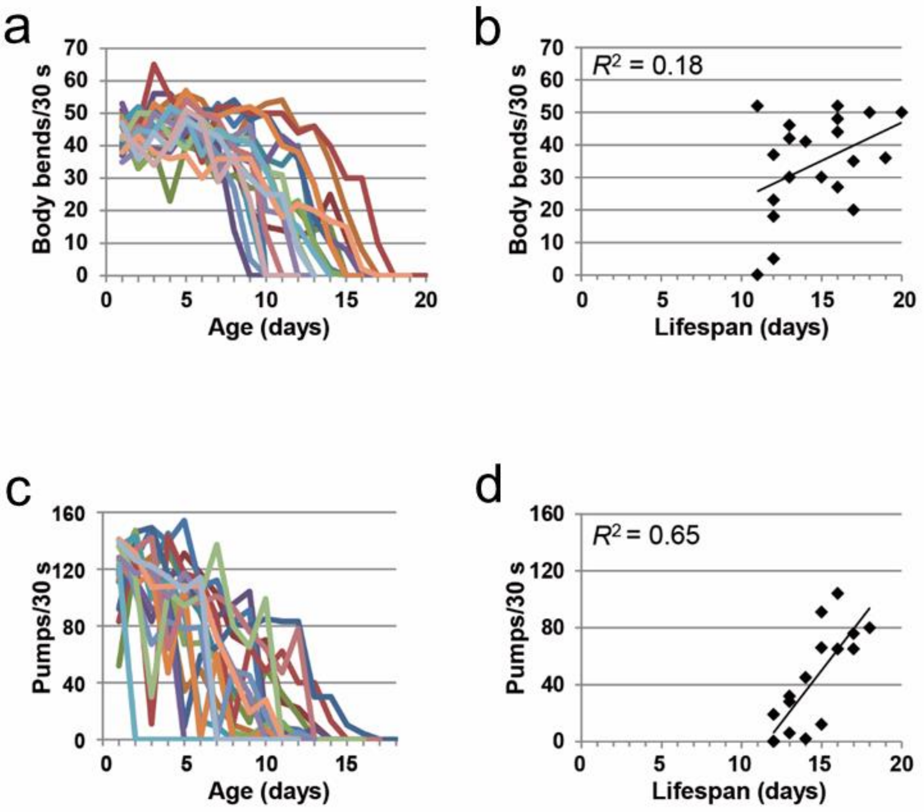

Supplementary Figure 5: Age-dependent changes in the thrashing and pharyngeal pumping. (**a**) Age-associated change of thrashing. Thrashing of each worm is noted as body bends/30 s. (**b**) Correlation between thrashing and lifespan of individual worms. Thrashing of individual worms was measured at Day 9 of adulthood and their lifespans were measured thereafter. R^2, coefficient of determination. *n* = 20. (**c**) Age-associated change of pharyngeal pumping rate. (**d**) Correlation between pharyngeal pumping and lifespan of individual worms. The pumping rates of individual worms were measured at Day 9 of adulthood and their lifespans were measured thereafter. R^2, coefficient of determination. *n* = 19. Note that, although the pharyngeal pumping rates showed a coefficient of determination value of 0.65, they showed highly irregular patterns along aging and thus do not serve as a good age-associated indicator.

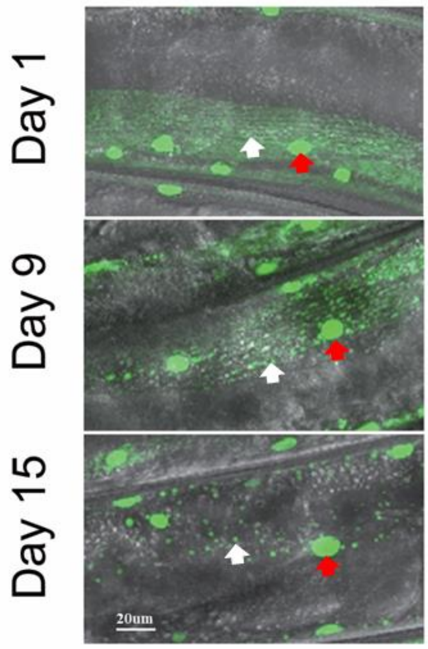

Supplementary Figure 6: Age-dependent changes of mitochondrial morphology in wild-type worms.

Top: mitochondria in the muscle cell of a worm at Day 1 of adulthood show tubular and intermediate morphology. Middle: fragmented mitochondria appear in a worm at Day 9 of adulthood. Bottom: mitochondria in a worm at Day 15 of adulthood are very fragmented with a few globular organelles. Colored green is the green fluorescence protein produced in mitochondria (white arrow) and nucleus (red arrow) in body wall muscles of PD4251 worms.

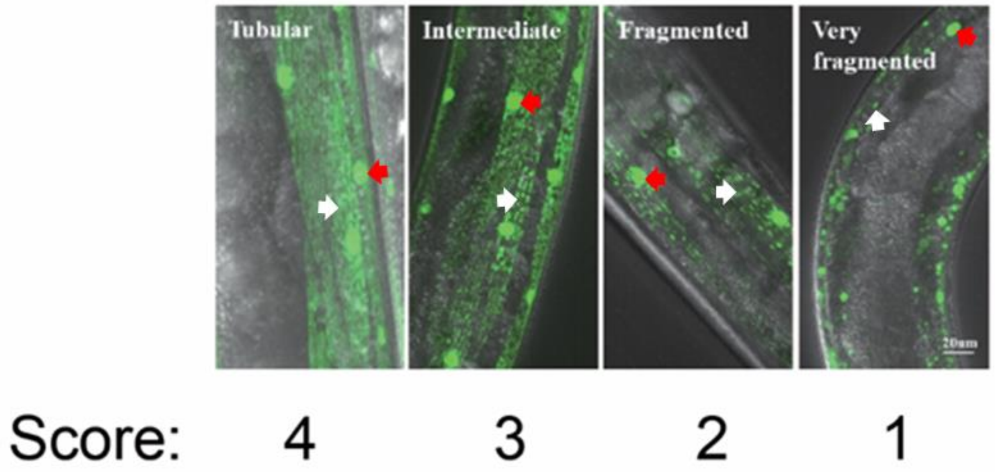

Supplementary Figure 7: Scoring of mitochondrial morphologies for mitochondrial integrity. To assay mitochondrial integrity, the scores of 1, 2, 3, and 4 were given to mitochondria with very fragmented, fragmented, intermediate, and tubular morphologies, respectively. Colored green is the green fluorescence protein produced in mitochondria (white arrow) and nucleus (red arrow) in body wall muscles of PD4251 worms.

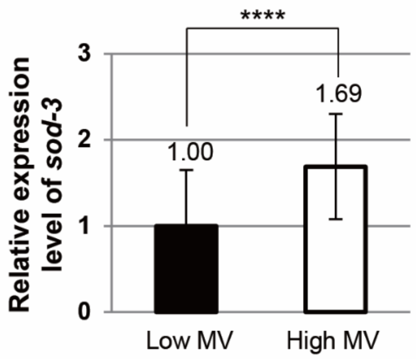

Supplementary Figure 8: Correlation of *sod-3* gene expression with MV in the low and high MV groups at Day 9 of adulthood. Significance was determined using a two-tailed, unpaired *t*-test. ****P<0.0001. Error bars represent s.d.

a.

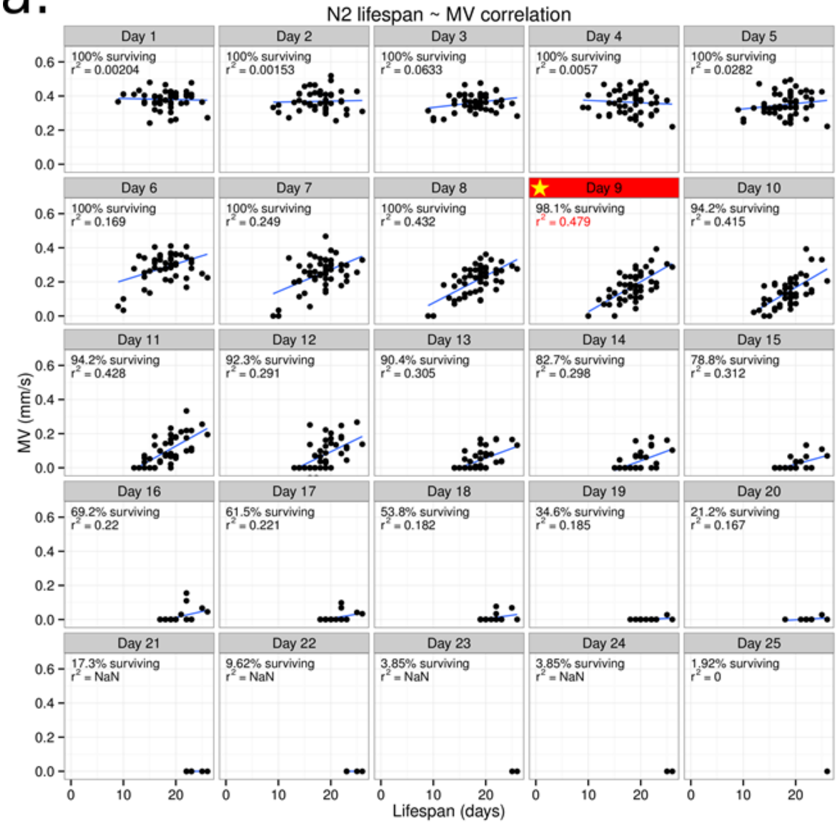
N2 lifespan ~ MV correlation
MV (mm/s)
Lifespan (days)
Day 1 100% surviving r^2 = 0.00204
Day 2 100% surviving r^2 = 0.00153
Day 3 100% surviving r^2 = 0.0633
Day 4 100% surviving r^2 = 0.0057
Day 5 100% surviving r^2 = 0.0282
Day 6 100% surviving r^2 = 0.169
Day 7 100% surviving r^2 = 0.249
Day 8 100% surviving r^2 = 0.432
Day 9 98.1% surviving r^2 = 0.479
Day 10 94.2% surviving r^2 = 0.415
Day 11 94.2% surviving r^2 = 0.428
Day 12 92.3% surviving r^2 = 0.291
Day 13 90.4% surviving r^2 = 0.305
Day 14 82.7% surviving r^2 = 0.298
Day 15 78.8% surviving r^2 = 0.312
Day 16 69.2% surviving r^2 = 0.22
Day 17 61.5% surviving r^2 = 0.221
Day 18 53.8% surviving r^2 = 0.182
Day 19 34.6% surviving r^2 = 0.185
Day 20 21.2% surviving r^2 = 0.167
Day 21 17.3% surviving r^2 = NaN
Day 22 9.62% surviving r^2 = NaN
Day 23 3.85% surviving r^2 = NaN
Day 24 3.85% surviving r^2 = NaN
Day 25 1.92% surviving r^2 = 0

b.

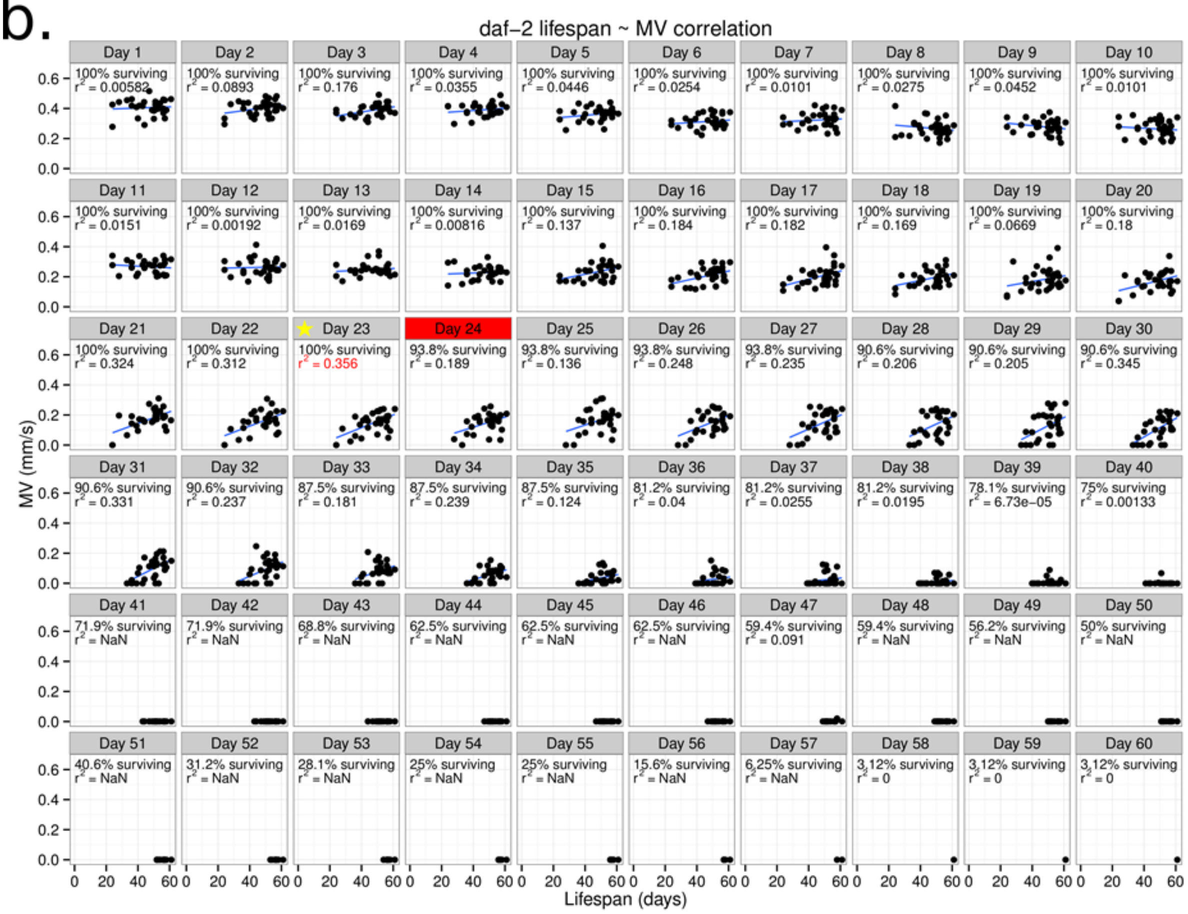
daf−2 lifespan ~ MV correlation
MV (mm/s)
Lifespan (days)
Day 1 100% surviving r^2 = 0.00582
Day 2 100% surviving r^2 = 0.0893
Day 3 100% surviving r^2 = 0.176
Day 4 100% surviving r^2 = 0.0355
Day 5 100% surviving r^2 = 0.0446
Day 6 100% surviving r^2 = 0.0254
Day 7 100% surviving r^2 = 0.0101
Day 8 100% surviving r^2 = 0.0275
Day 9 100% surviving r^2 = 0.0452
Day 10 100% surviving r^2 = 0.0101
Day 11 100% surviving r^2 = 0.0151
Day 12 100% surviving r^2 = 0.00192
Day 13 100% surviving r^2 = 0.0169
Day 14 100% surviving r^2 = 0.00816
Day 15 100% surviving r^2 = 0.137
Day 16 100% surviving r^2 = 0.184
Day 17 100% surviving r^2 = 0.182
Day 18 100% surviving r^2 = 0.169
Day 19 100% surviving r^2 = 0.0669
Day 20 100% surviving r^2 = 0.18
Day 21 100% surviving r^2 = 0.324
Day 22 100% surviving r^2 = 0.312
Day 23 100% surviving r^2 = 0.356
Day 24 93.8% surviving r^2 = 0.189
Day 25 93.8% surviving r^2 = 0.136
Day 26 93.8% surviving r^2 = 0.248
Day 27 93.8% surviving r^2 = 0.235
Day 28 90.6% surviving r^2 = 0.206
Day 29 90.6% surviving r^2 = 0.205
Day 30 90.6% surviving r^2 = 0.345
Day 31 90.6% surviving r^2 = 0.331
Day 32 90.6% surviving r^2 = 0.237
Day 33 87.5% surviving r^2 = 0.181
Day 34 87.5% surviving r^2 = 0.239
Day 35 87.5% surviving r^2 = 0.124
Day 36 81.2% surviving r^2 = 0.04
Day 37 81.2% surviving r^2 = 0.0255
Day 38 81.2% surviving r^2 = 0.0195
Day 39 78.1% surviving r^2 = 6.73e−05
Day 40 75% surviving r^2 = 0.00133
Day 41 71.9% surviving r^2 = NaN
Day 42 71.9% surviving r^2 = NaN
Day 43 68.8% surviving r^2 = NaN
Day 44 62.5% surviving r^2 = NaN
Day 45 62.5% surviving r^2 = NaN
Day 46 62.5% surviving r^2 = NaN
Day 47 59.4% surviving r^2 = 0.091
Day 48 59.4% surviving r^2 = NaN
Day 49 56.2% surviving r^2 = NaN
Day 50 50% surviving r^2 = NaN
Day 51 40.6% surviving r^2 = NaN
Day 52 31.2% surviving r^2 = NaN
Day 53 28.1% surviving r^2 = NaN
Day 54 25% surviving r^2 = NaN
Day 55 25% surviving r^2 = NaN
Day 56 15.6% surviving r^2 = NaN
Day 57 6.25% surviving r^2 = NaN
Day 58 3.12% surviving r^2 = 0
Day 59 3.12% surviving r^2 = 0
Day 60 3.12% surviving r^2 = 0

c.

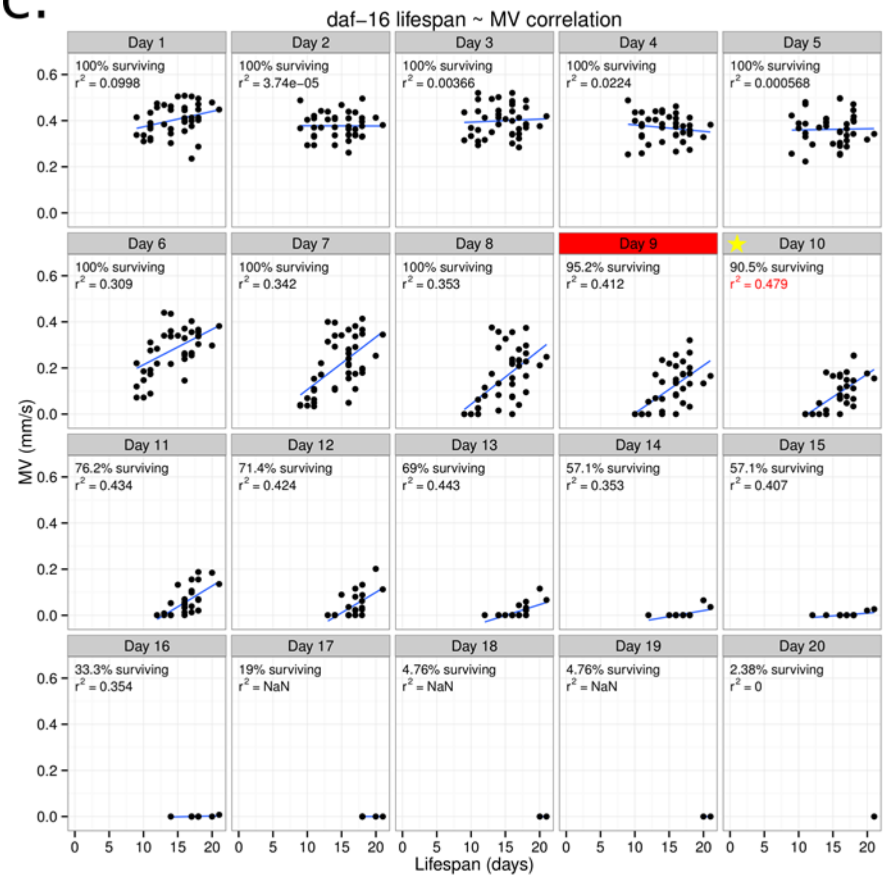

Supplementary Figure 9: Correlation between MV and lifespan for a cohort of worms tracked longitudinally through their lifespan.

The day on which the first worm of the cohort dies is highlighted in red, and the day on which MV best correlates with lifespan is denoted with a star. In all cases tested, the best correlation between MV and lifespan occurs within a day of the first death of the cohort. (**a**) N2 worms tracked in Fig. 1a. The best correlation between MV and lifespan occurs on day 9, which is also when the first worm of the cohort dies. (**b**) *daf-2(e1370)* worms tracked in Fig. 2b. The best correlation between MV and lifespan occurs on day 23, the day before the first worm of the cohort dies. (**c**) *daf-16(mu86)* worms tracked in Fig. 2c. The best correlation occurs on day 10, the day after the first worm of the cohort dies.

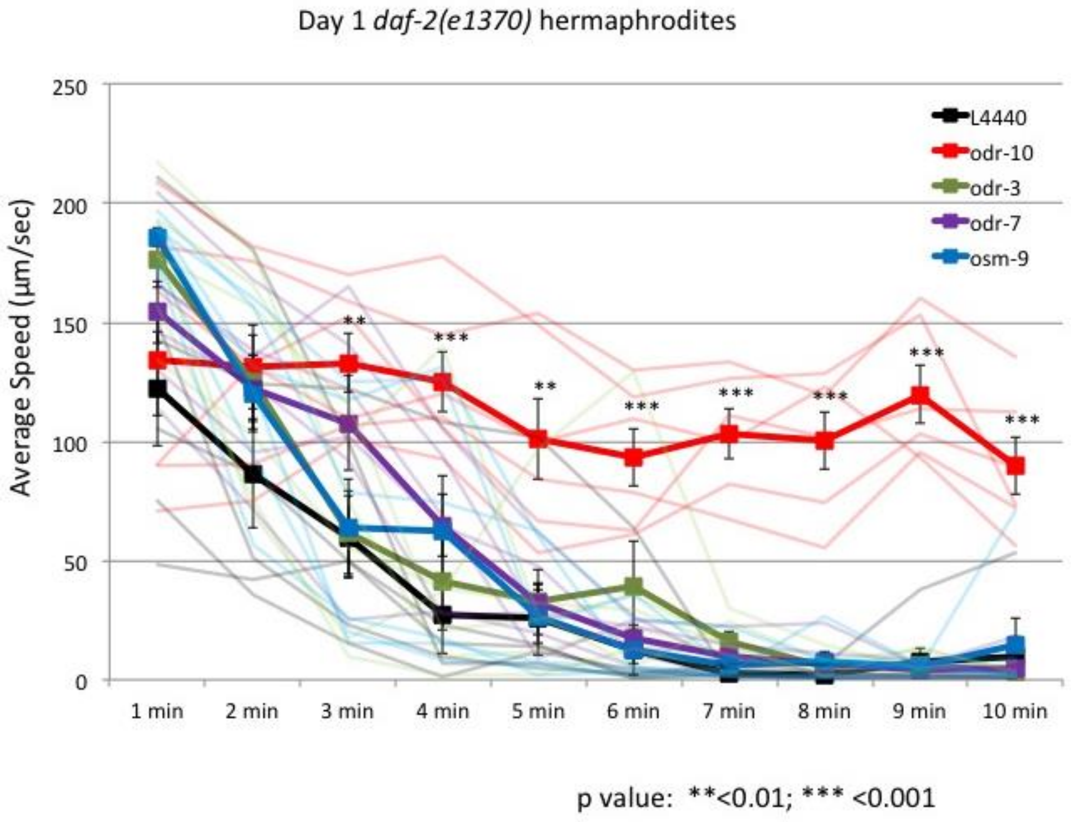

Supplementary Figure 10: On-bacteria motility assay. *daf-2(e1370)* worms after being raised on L4440 (control), *odr-3, odr-7*, or *osm-9* RNAi slow down soon after being placed onto bacteria, while *daf-2(e1370)* worms treated with *odr-10* RNAi (red) maintain a higher speed over the entire 10 min of the assay.

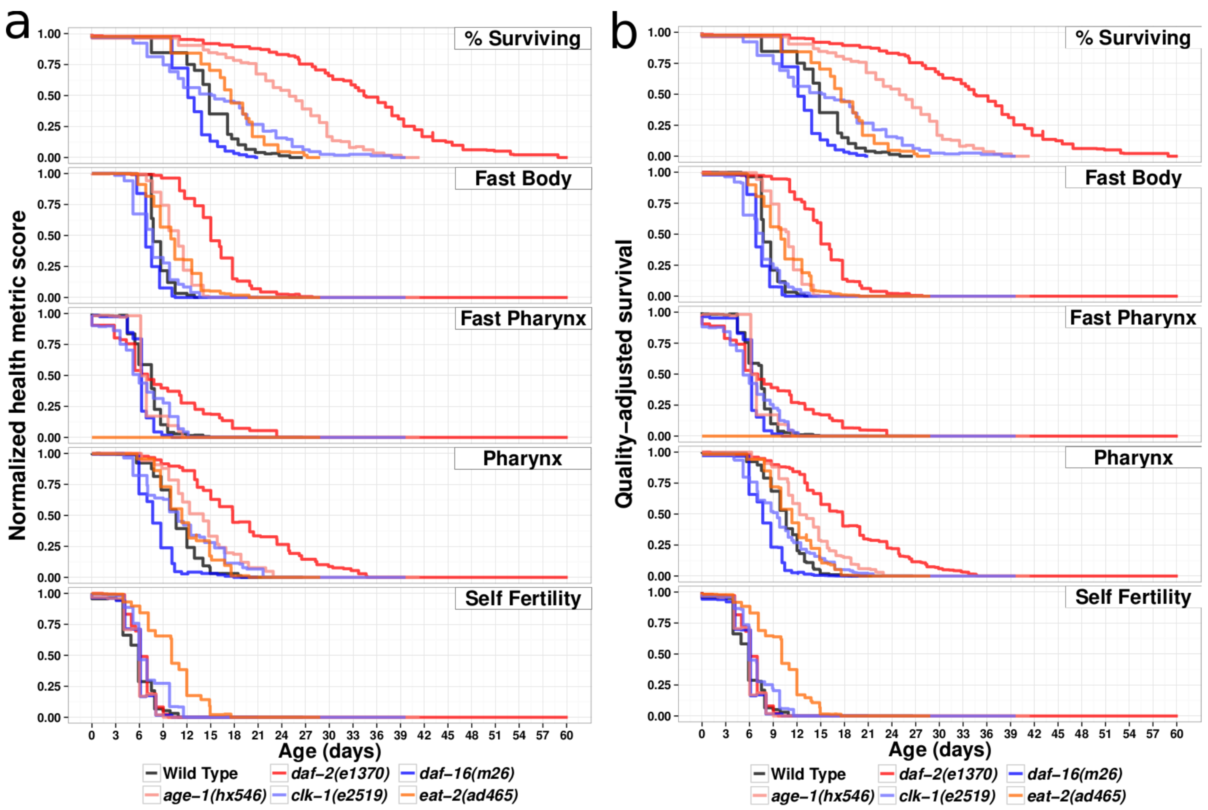
a
b
% Surviving
Fast Body
Fast Pharynx
Pharynx
Self Fertility
Normalized health metric score
Quality-adjusted survival
Age (days)
Wild Type
daf-2(e1370)
daf-16(m26)
age-1(hx546)
clk-1(e2519)
eat-2(ad465)

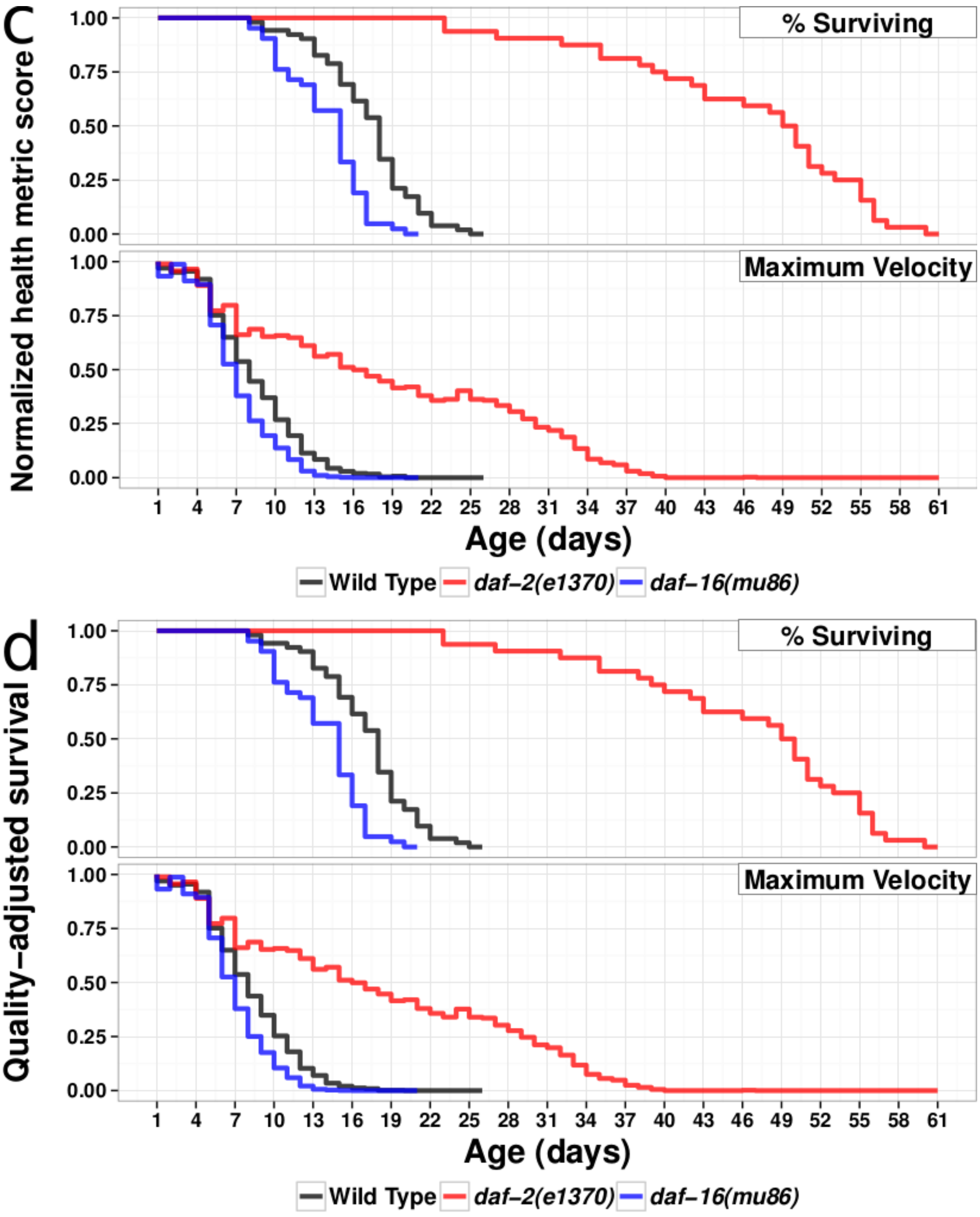
c
Normalized health metric score
% Surviving
Maximum Velocity
1.00
0.75
0.50
0.25
0.00
1 4 7 10 13 16 19 22 25 28 31 34 37 40 43 46 49 52 55 58 61
Age (days)
Wild Type
daf-2(e1370)
daf-16(mu86)
d
Quality-adjusted survival
% Surviving
Maximum Velocity
1.00
0.75
0.50
0.25
0.00
1 4 7 10 13 16 19 22 25 28 31 34 37 40 43 46 49 52 55 58 61
Age (days)
Wild Type
daf-2(e1370)
daf-16(mu86)

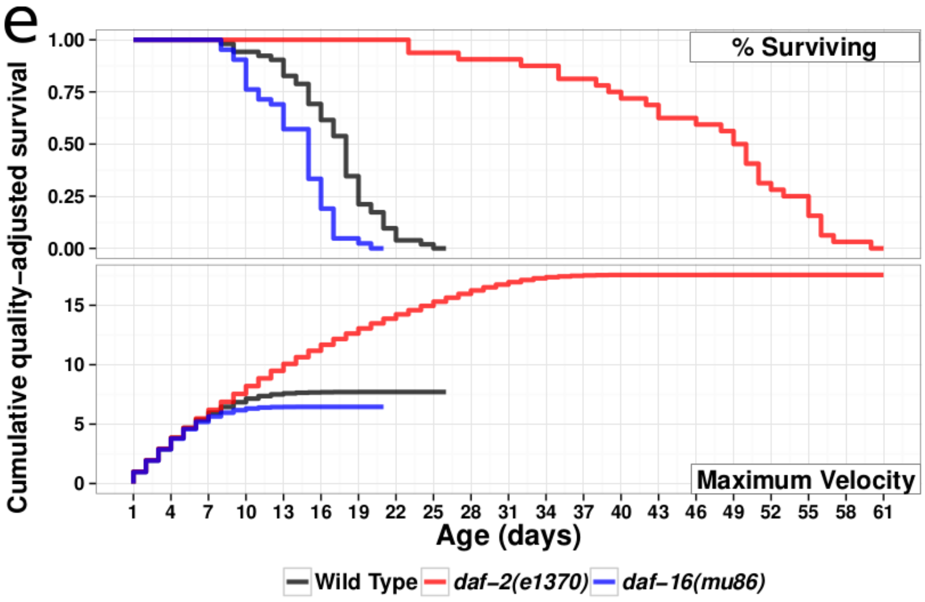

Supplementary Figure 11: Additional quality-adjusted health metrics

daf-2(e1370) mutants are healthier and have higher quality lives as measured in previous surveys of healthspan. (**a**) Quality-adjusted lifespan curves for the health categories, as scored by Huang et al.,[1] with categories separated by the health categories used in the study. (**b**) The Quality-adjusted lifespan curve is the survival rate multiplied by the health measurement, normalized to the maximum value attained by the wild type. From our own data on MV with age, we find *daf-2* mutants have higher quality life than wild type. (**c**), Quality-adjusted lifespan curves for MV as measured in this study. (**d**) Cumulative area under curve of the quality-adjusted MV lifespan, indicating higher total quality for *daf-2* mutants, and lower total quality for *daf-16(mu86)* mutants as compared to wild type. (**e**) Cumulative area under curve of the quality-adjusted MV.

Supplementary Reference

1. Huang C, Xiong CJ, Kornfeld K. Measurements of age-related changes of physiological processes that predict lifespan of Caenorhabditis elegans. *Proc Natl Acad Sci U S A* **101**, 8084-8089 (2004).
